# Hopeless Neuroma—The Neurotized Free Flap Tissue Augmentation as Salvage Therapy—A Concept and Clinical Demonstration

**DOI:** 10.3390/jpm13020313

**Published:** 2023-02-10

**Authors:** Martin Aman, Julia J. Glaser, Arne H. Boecker, Mirjam Thielen, Amr Eisa, Amir K. Bigdeli, Emre Gazyakan, Ulrich Kneser, Leila Harhaus

**Affiliations:** Department of Hand, Plastic and Reconstructive Surgery, Burn Center, BG Trauma Center Ludwigshafen, Department of Hand and Plastic Surgery, University of Heidelberg, 67071 Ludwigshafen, Germany

**Keywords:** reconstructive surgery, reconstructive microsurgery, flap surgery, plastic surgery, neuroma, neurotization

## Abstract

Therapy-resistant neuroma pain is a devastating condition for patients and surgeons. Although various methods are described to surgically deal with neuromas, some discontinuity and stump neuroma therapies have anatomical limitations. It is widely known that a neurotizable target for axon ingrowth is beneficial for dealing with neuromas. The nerve needs “something to do”. Furthermore, sufficient soft tissue coverage plays a major role in sufficient neuroma therapy. We aimed, therefore, to demonstrate our approach for therapy of resistant neuromas with insufficient tissue coverage using free flaps, which are sensory neurotized via anatomical constant branches. The central idea is to provide a new target, a new “to do” for the painful mislead axons, as well as an augmentation of deficient soft tissues. As indication is key, we furthermore demonstrate clinical cases and common neurotizable workhorse flaps.

## 1. Background

Painful neuroma formation after sustaining a peripheral nerve injury is a devastating condition. Patients suffer from electrical or burning pain, amplified by even the lightest touch of the injured area.

The exact prevalence of neuroma formation is unknown; the current literature describes rates from 1% up to 10% of patients sustaining a peripheral nerve injury [1,2].

Treatment options range from conservative to surgical treatment. Here, multiple techniques are described, from simple neuroma excision to transferring the nerve in various tissues, such as veins, muscles, bone or neighbored nerves [3]. However, improvement after surgical therapy can only be achieved in 77% [4]. Due to a high recurrence rate, the reoperation rates range between 20% and 27%, with a decreasing success rate for each additional operation [5,6]. So far, no treatment has been demonstrated as superior, although it seems to be essential to create a new neurotizable target to mislead axons by transferring the nerve stump to muscle tissue or another nerve [7].

Therefore, new techniques such as targeted muscle reinnervation (TMR) and regenerative peripheral nerve interfaces (RPNI) have been developed and show promising results [7,8]. Their underlying concept of “giving the nerve a new target” seems to be the key factor of success compared to the traditional techniques. However, these techniques are established for amputation cases and stump neuroma, not for a neuroma in a preserved extremity.

Besides a new target for regenerating axons, sufficient soft (gliding) tissue coverage of painful neuroma sites has been described as fundamental by Millesi et al. [9].

With this knowledge, the modern reconstructive surgeon can combine treatment strategies out of the armamentarium of neuroma treatment and free flap surgery to develop a new treatment strategy for severe and therapy-resistant cases of discontinuity neuroma with insufficient soft tissue coverage.

### 1.1. General Neuroma Considerations

Neuromas are thickened portions of damaged nerves that have uncoordinated axons sprouting surrounded by scar tissue. They consist of axons, Schwann cells, and fibroblasts that are perineurally attached to the surrounding tissue [8]. 

Typically, symptomatic neuromas lead to loss of function or (severe) pain. It is noteworthy here that not all neuromas are symptomatic. Any nerve injury results in a scarring of the nerve, similar to a neuroma. The pain manifests itself in a variety of ways: it can trigger a pain stimulus with a mechanical stimulus or spontaneously. A phantom pain or a central imprint in the pain memory can also lead to painful events.

Physiologically, nerves have a certain sliding bearing; for example, the median nerve has a sliding bearing of 2–3 cm [9]. If there is a movement here in which the nerve cannot slide due to internal scarring, electrifying pain can follow.

Classic approaches such as neuroma excision or conservative therapy approaches have been in the foreground so far. In recent years, however, peripheral nerve surgery has been expanded to include treatment options such as nerve reconstruction using allografts/autografts, end-to-side neurorrhaphy, TMR (=Targeted Muscle Reinnervation) and RPNI (=Regenerative Peripheral Nerve Interface). These treatment options are only feasible for terminal neuromas as otherwise, function would be lost. Furthermore, they face the aforementioned anatomical limitations.

### 1.2. Principle of the New Target Transfer

The idea is to transfer a microvascular fasciocutaneous free flap, including a cutaneous nerve, to the injured nerve, where the nerve branch can be coapted with the injured nerve. Hereby, the transferred flap can serve as a new target and allow the nerve to grow in and find sensory end organs. This technique meets the requirements of a “new target theory” even for a chronic neuroma in a preserved limb and offers sufficient soft tissue coverage [10].

Especially for areas of the body, which only provide thin soft tissue coverage of superficial nerves and limited expendable recipients for nerve transfers (e.g., dorsal hand, superficial radial nerve branch or the knee, infrapatellar branch), this technique can provide a promising solution. Thus, especially these areas are prone to painful neuroma development after nerve injury [11].

We aimed, therefore, to describe our approach for patients with severe, therapy-resistant neuroma pain by creating a new nerve target with an expendable recipient nerve combined with free flap autologous soft tissue augmentation as salvage therapy.

## 2. Indications

**Figure 1:**
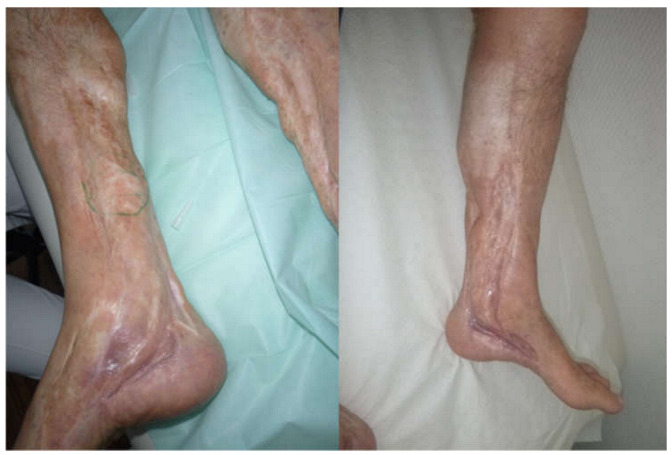
Example of a potential indication for a neurotized flap. Patient is suffering from severe tarsal tunnel syndrome and has undergone multiple operations, leaving a scarred and hardly covered environment for the nerve, resulting in severe neuroma pain.

The method can be used as a salvage therapy for recurrent neuroma after other surgical attempts have failed. We recommend prior, less-invasive surgical interventions such as neuroma excision and nerve transfer or nerve reconstruction if possible. This might not always be achievable in some areas of the body, such as the infrapatellar branch of the saphenous nerve with the above-mentioned anatomical limitations (Figure 1). 

Another indication is a pre-operated area with insufficient soft tissue coverage and superficial neuroma. By expansion of gliding tissue, the nerve has a thicker layer of coverage, preventing external irritation [9,10].

## 3. Neurotizable Workhorse Flaps

Several flaps can be used to increase the gliding tissue of the nerve and to create a new target for axon ingrowth. These are determined by an anatomically consistent cutaneous nerve branch and a fasciocutaneous flap providing the best gliding tissue. The sensory donor site morbidity in all flaps is usually neglectable as the area of the transferred flap only leaves a small area around the scar with sensory deprivation.

**Figure 2:**
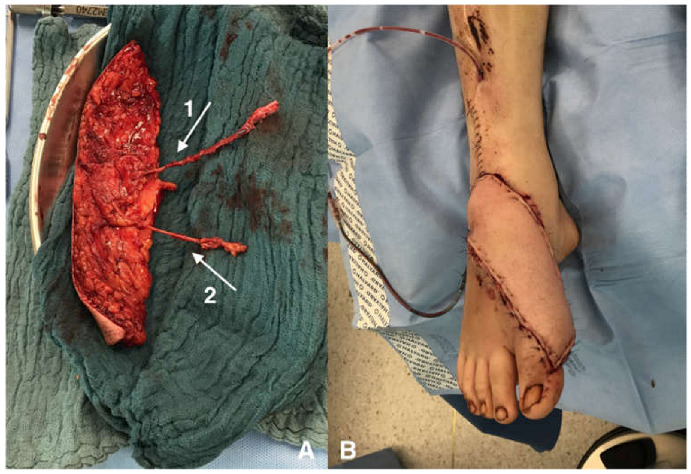
Example of an ALT flap, which can be used for neuroma salvage. (**A**) shows the cutaneous branch (2) besides the vascular pedicle (1), which has excellent length and diameter for providing a good recipient nerve as a new neurotizable target. (**B**) shows the application on the dorsal foot and ankle region, where only a thin soft tissue layer covers the superficial nerves.

The anterolateral thigh (ALT) flap is one of the workhorse flaps in reconstructive microsurgery and, in most cases, the preferred neurotizable flap of the authors. The perforator-based blood supply is on the descending branch of the lateral circumflex femoral artery. Nerve supply can be harvested from the lateral cutaneous femoral nerve, which pierces the fascia about 10 cm below the inguinal ligament. It is usually a thin flap, providing excellent soft tissue coverage and gliding capacity through its fascia, and can be time-efficiently harvested without large donor site morbidity (Figure 2). 

**Figure 3:**
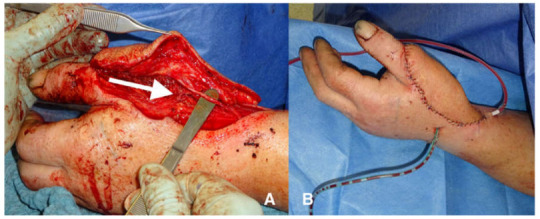
Example of a lateral arm flap (LAF). (**A**) shows the constant cutaneous branch with good diameter for neurotization. (**B**) demonstrates not only the application at wrist level but furthermore a thin and appealing appearance of the flap.

Another thin flap with consistent neurovascular anatomy is the lateral arm flap (LAF). Based on the radial collateral artery, a nerve branch of the lower brachial cutaneous nerve can be harvested for nerve transfer. Again, donor site morbidity is small and primary closure of the donor site should be easily achievable [12] (Figure 3). 

The radial forearm flap is a thin flap, ideally for delicate reconstruction with a large and reliable blood supply by the radial artery and a sensory branch of the lateral antebrachial cutaneous nerve. When harvesting this flap, considerable donor site morbidity of the radial artery and potential split skin grafting should be considered and opposed to the indication of neuroma salvage and tissue augmentation [13].

The deep inferior epigastric perforator flap (DIEP) is also described to be available for neurotization. The nerve branches of intercostal nerves are described as following the arteriovenous perforators [14]. Yet, this flap is usually bulkier than the above-described options, and applications for distal extremity reconstruction are, therefore, limited.

## 4. Perioperative Management

Preoperatively, flap perforators are visualized with duplex ultrasound. Although high-resolution ultrasound also allows visualization of sensory nerves within the flap, it is not necessary in the majority of cases due to the constant anatomy. 

In contrast, at the recipient site, preoperative visualization of the exact neuroma location is useful for operative planning, although the anatomic correlation to the Hoffmann–Tinel sign is very high. This is also relevant for medicolegal considerations and planning of operations in patients who underwent prior surgery. This should be achieved with ultrasound or MRI. We furthermore recommend standard flap preparations such as preoperative blood and coagulation tests and potential transfusion preparation.

Postoperatively all patients are treated according to established standards for free flap surgery with antithrombotic therapy, resting, and perfusion checkup routine for the first five postoperative days, which is then followed by flap training and tissue conditioning. Additionally, it is important to cover the neuropathic pain aspects. Therefore, we started placing a local pain catheter prior to surgery on the affected nerve to block upcoming signals from the nerve during surgery and neurotomy, potentially triggering central pain perception.

Furthermore, local pain catheter placement and administration of constant local anesthesia are performed for the first days, always in close collaboration with pain specialists. During inpatient treatment, regular consultations with pain specialists and psychological treatment are performed to treat central pain memory. After dismission, we encourage the fitting of compression stockings and regular outpatient follow-up visits.

## 5. Clinical Application

**Figure 4:**
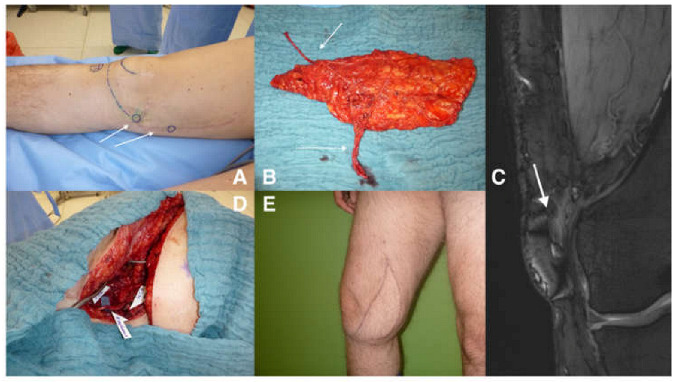
Clinical demonstration of the procedure. (**A**) shows the areas with positive Hoffmann–Tinel sign after arthroscopy of the knee. The neuroma formation was also detected in MRI scans (**C**). (**B**) shows the cutaneous branch beside the vascular pedicle, which was anastomosed to the femoral artery and greater saphenous vein (**D**), as well as coaptated with the remaining saphenous nerve. Final result is not only functional but also cosmetically appealing for the patient (**E**).

To obtain a better understanding of the procedure, we present its application on an exemplary patient who underwent an arthroscopy of the knee and suffered a nerve injury of the infrapatellar branch of the saphenous nerve. Over the course of several years of pain with excessive pain medication intake (preoperative medicaments: gabapentin 1.5 g/d, oxycodone 40 mg/d, ibuprofen 1.8 g/d, metamizole 4 g/d), local anesthetics and botox injections (200IE xeomin injection every 3–4 months) and several surgical procedures such as neurolysis, neuroma resection and coagulation as well as neuroma resection and placement in nerve cap, the patient still suffered from a pain of 8/10 on the visual analog scale. He especially experienced severe pain attacks even with the slightest touch of the area of the neuroma. After multiple detailed discussions with the patient and counseling, we transferred an ALT flap to enhance the thin soft tissue, which was massively scarred from the previous surgeries. The ALT flap was raised in the epifascial layer, and a cutaneous branch of the lateral femoral cutaneous nerve was dissected. Neurorrhaphy of the cutaneous branch to the saphenous nerve was performed in order to create a new target for sensory reinnervation. Postoperatively, no adverse events were noticed. During follow-up (of 24 months), pain reduction was significant, although the patient was never completely pain-free (VAS 2–3). Pain attacks could be reduced via tissue augmentation, and the hypersensitivity of the area decreased, leading to an increased quality of life. Additionally, pain medication was decreased tremendously, and the patient is now able to live without opioids and a quarter of the initial gabapentin dose. One year after flap surgery, a refinement surgery was performed to further reduce the volume of the ALT flap (Figure 4). 

## 6. Discussion

The development of chronic pain after peripheral nerve injuries is attributed to changes at all processing levels of the somatosensory system. An influence of many different factors, such as psychosocial factors, surgical techniques, perioperative pain medication, etc., is assumed [15,16]. Chronic neuropathic pain is generally considered difficult to treat [17]. It is a very distressing condition for those affected, which is often associated with sleep disorders, depressive moods, and social withdrawal. The consequences are long downtimes and a high level of medical utilization [18].

The transposition of the nerve stump into adjacent muscle tissue is a standard procedure in neuroma therapy and is intended to move the sensitive stump deeper and, at the same time, protect it from external pressure with a good soft tissue layer. This common and frequently performed technique has been described for almost all lesion levels and nerve types. Larger studies describe good pain reduction in 64 to 82% of cases [19,20]. However, the recurrent traction that the muscle exerts on the implanted nerve during activity can be problematic. This technique is also distinguished from neurotization, since the nerve stump is implanted in non-denervated muscle tissue and, thus, there are no “free” motor end plates that could serve as a target for the nerve. The muscle serves only as mechanical padding.

Alternatively, the principle of targeted muscle reinnervation (TMR) consists of relocating the affected nerve stumps to a motor nerve branch that leads to a muscle that is still intact. On the one hand, this muscle is initially denervated via a primary end-to-end coaptation. On the other hand, the sprouting axons can be routed into this motor nerve branch and then encounter briefly denervated muscle tissue or motor end plates, which they can reinnervate. A very low neuroma rate and a reduction in phantom pain were observed as effects [3,21].

Derived from the considerations regarding TMR, bringing a new target organ into the site which can be reinnervated by the nerve stump seems to fulfill this concept even without muscle targets.

In this way, free adipocutaneous flaps, including the associated cutaneous nerve, can be transferred as a sensory target. After neuroma resection, the painful nerve stump can be connected to the cutaneous nerve via a primary end-to-end coaptation, so that the axons can sprout into the associated dermatome, namely the flap. This has the further advantage of being time-independent, as preserved sensory end organs are transferred, independent of denervation time. At the same time, a stable, sufficient soft tissue coverage can be achieved with the flap, which is a crucial prerequisite for recurrence prevention.

We therefore adapted the previously described concept of soft tissue augmentation for neuroma coverage with the concept of creating a new target for neurotization to provide a target for axonal ingrowth, reducing neuroma formation [9,22,23].

We prefer the ALT flap due to its constant anatomy and variability [24,25]. The lateral arm flap hereby is another flap with reliable nerve anatomy. As this procedure is mainly used as salvage, only flaps with reliable anatomy that can be performed by the surgeon should be chosen.

In general, this concept is useful to decrease pain and especially severe pain attacks induced by even the slightest touch. This is a huge improvement in the quality of life of patients. We also experienced that most patients do not fully recover from pain, leaving a generalized pain in the previous area of the neuroma. Hereby, central pain memory plays a major role and should be addressed by additional therapy, such as mirror therapy or other therapies that support the cortical reorganization of the somatosensory (S1) cortex [26].

## 7. Conclusions

We present an alternative therapy option for severe and therapy-resistant neuroma pain in areas with insufficient soft tissue coverage and no available expendable donor nerves for selective nerve transfers. Hereby, a new neurotizable target is created to allow for axonal ingrowth, decreasing neuroma formation. Although most patients do not become completely pain-free due to the central pain memory of long preexisting neuropathic pain, they report significant improvement in quality of life.

## Data Availability

Not applicable.
